# Pain Assessment Using Virtual Reality Facemask During Bone Marrow Aspiration: Prospective Study Including Propensity-Matched Analysis

**DOI:** 10.2196/33221

**Published:** 2022-10-12

**Authors:** Lou Soret, Nicolas Gendron, Nadia Rivet, Richard Chocron, Laure Macraigne, Darless Clausse, Bernard Cholley, Pascale Gaussem, David M Smadja, Luc Darnige

**Affiliations:** 1 Innovative Therapies in Haemostasis, INSERM Université Paris Cité Paris France; 2 Hematology department Assistance Publique Hôpitaux de Paris-Centre-Université de Paris Paris France; 3 Biosurgical research lab Carpentier Foundation Assistance Publique Hôpitaux de Paris-Centre-Université de Paris Paris France; 4 PARCC, INSERM Université Paris Cité Paris France; 5 Emergency Medicine Department Georges Pompidou European Hospital Paris France; 6 Department of Anesthesia and Intensive Care Georges Pompidou European Hospital Paris France; 7 Biosurgical Research Lab Carpentier Foundation Georges Pompidou European Hospital Paris France

**Keywords:** bone marrow aspiration, pain assessment, virtual reality facemask, anxiety, hematology, virtual reality, VR, haematology, haematological, hematological, hematological disorder, pain, pain scale, medical procedure, bone marrow, facemask, diagnosis, monitoring, anxiety

## Abstract

**Background:**

Bone marrow aspiration (BMA) is a medical procedure necessary to the diagnosis and monitoring of patients with hematological or nonhematological disorders. This procedure is considered painful, and patients are generally anxious before and during BMA.

**Objective:**

This study assesses the effect of immersive virtual reality on pain during BMA.

**Methods:**

This observational prospective and monocentric study enrolled 105 consecutive patients who underwent sternal BMA with lidocaine anesthesia. The study was carried on during 2 periods. First, virtual reality facemask (VRF) was proposed to all patients in the absence of exclusion criteria. During the second period, BMA was performed without the VRF. For all patients, pain intensity after the procedure was assessed using a 10-point numerical pain rating scale (NPRS). All analyses were performed on propensity score–matched cohort (with or without VRF) to evaluate efficacy on NRPS levels.

**Results:**

The final matched cohort included 12 patients in the VRF group and 24 in the control group. No difference in anxiety level before BMA evaluated by the patient and by the operator was observed between groups (*P*=.71 and .42 respectively). No difference of NPRS was observed using VRF when compared to control group (median NPRS 3.8, IQR 2.0-6.3 vs 3.0, IQR 1.9-3.0, respectively; *P*=.09).

**Conclusions:**

Our study did not prove the efficacy of VRF to reduce pain during BMA.

## Introduction

Bone marrow aspiration (BMA) is a standard procedure for diagnosis, staging, prognosis, and follow-up response to treatment of numerous hematologic and some nonhematologic diseases. BMA is generally carried out in adult patients without general anesthesia [1] and may be performed at different puncture sites. In France, the most common site of aspiration in adults [2] is the sternal manubrium because it is more accessible than iliac crest and may be safely performed in patients receiving anticoagulant treatment. Whatever the site of aspiration, BMA is still considered a painful procedure, and standardization of pain prevention remains a major issue. Moreover, the increase in anxiety in a clinical environment can worsen the perception of pain [3]. In a previous study, we showed that despite local anesthesia, pain scores obtained using a numerical pain rating scale (NPRS) still ranged between 2.8 and 3.5 [4]. Whatever the puncture site, the patient needs to be reassured and well informed regarding the BMA procedure to decrease anxiety and pain. Indeed, in our previous cohort, more than half of the patients were anxious or very anxious before BMA, and anxiety was found to be a major predictor of pain during the procedure. The aim of this study was to explore the effects of immersive virtual reality on BMA-associated pain scores. Indeed, this technique uses multisensory stimulation to provoke patient’s immersion in a virtual environment and a state of hypnosis, which is used to facilitate anxiolysis and analgesia during some procedures [5]. Many studies demonstrated a significant reduction in pain or a reduction in procedural anxiety [6] using a virtual reality facemask (VRF). Therefore, we conducted an observational prospective and monocentric study to compare the effects of VRF on anxiety and pain in patients undergoing sternal BMA with lidocaine anesthesia.

## Methods

### Ethics Approval

All the patients included were informed of the research protocol by letter, allowing them to express their opposition to the use of their data, according to French legislation and the institutional review board. The study was performed in accordance with the Declaration of Helsinki and authorized by the French Data Protection Agency (CNIL-1922081), and each patient signed consent.

### Overview

This observational prospective and monocentric study enrolled consecutive patients who underwent sternal BMA with 1% lidocaine anesthesia for all patients and assessed pain during this procedure. All adult patients requiring sternal BMA between December 2019 and December 2020 were enrolled at the University Hôpital Européen Georges Pompidou (Assistance Publique-Hôpitaux de Paris, France).

**Figure 1:**
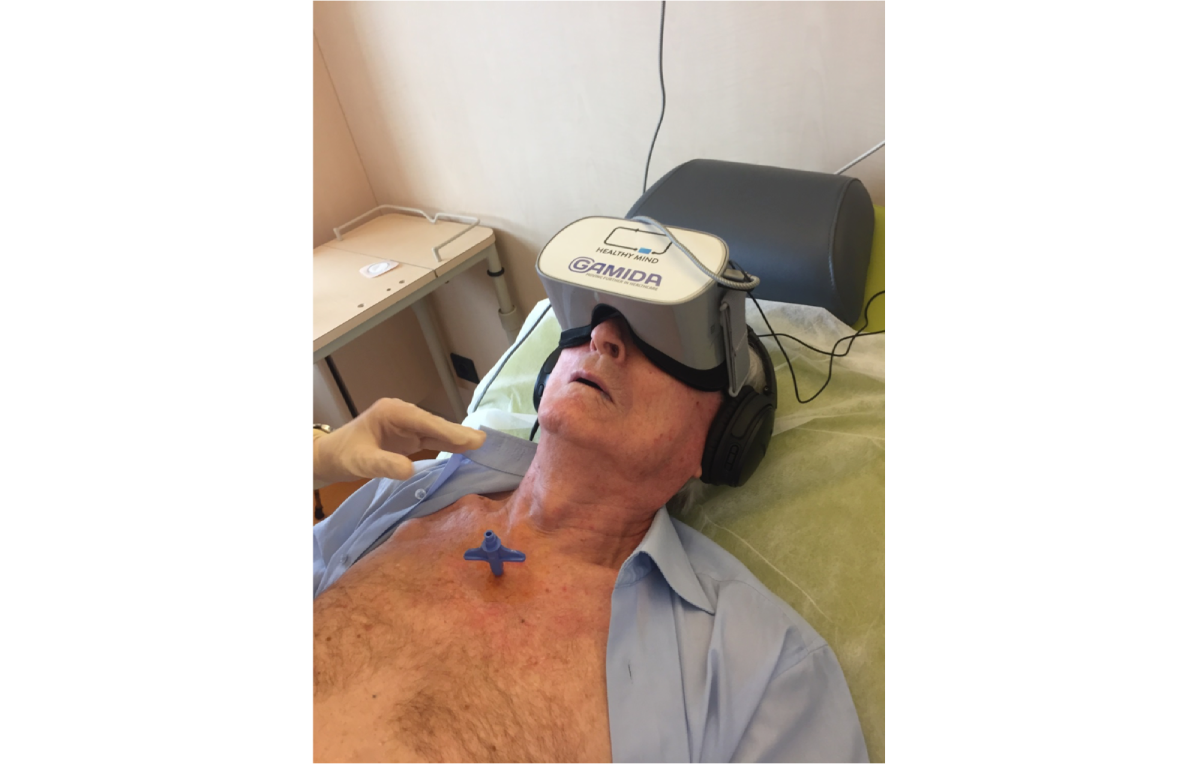
Patient wearing virtual reality facemask during sternal bone marrow aspiration.

The study was conducted during 2 periods. During the first period, immersive virtual reality using VRF was proposed to all patients (Figure 1). The VRF medical device was an Oculus Go helmet (Healthy Mind) consisting in a 3D video and audio headset, associated with virtual reality software. Patients were offered to choose 1 out of 3 relaxing environments (Zen garden, forest, or beach). During the second period, BMA was performed without VRF. Indeed, the COVID-19 pandemic did not allow us to use the VRF because of the risk of SARS-CoV-2 infection between patients. For both study periods, exclusion criteria were patient refusal, cognitive disorders, deep sedation, or language barrier. Patients were also excluded if they received any pharmacologic type of premedication or forms of analgesia other than subcutaneous lidocaine, such as a patch of local anesthetic or if they were offered to inhale nitrous oxide/oxygen gas premix (50%/50%).

For all patients, the same questionnaire was used, as previously described [4]. Briefly, this questionnaire included the following two assessments: (1) assessment of pain intensity following the procedurepatients were asked to quantify their pain intensity during BMA using a 10-point NPRS for which a score of 0 indicates no pain and a score of 10 indicates the worst imaginable pain; and (2) assessment of the patient’s anxiety before the procedurepatients were classified as nonanxious, anxious, or very anxious, both according to themselves and by the operator.

### Statistical Analysis

Since it has been reported in the literature that age and sex influence pain level during BMA [7-9], and to reduce confounding biases, we used propensity score method based on logistic regression to match patients with VRF with patients without VRF on sex and age using a 1:2 ratio. The matching created a balanced data set allowing comparison. In univariate analysis, continuous and categorical data were respectively expressed as median IQR (25th to 75th percentile) and as frequencies and percentages and compared using Mann-Whitney-Wilcoxon test and Fisher exact test. Statistical analysis was performed using R studio software, including R version 3.6.3 (R Development Core Team).

## Results

**Figure 2:**
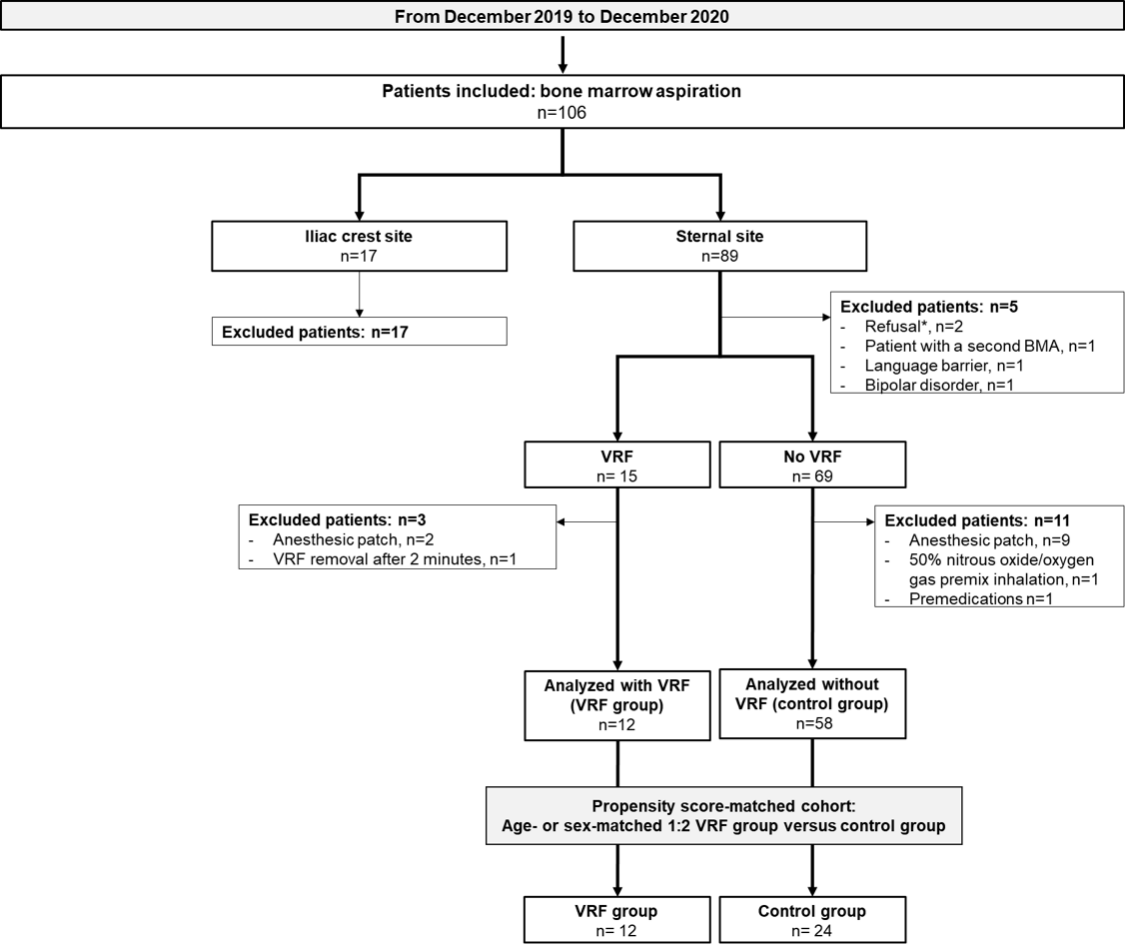
Patient flowchart. VRF: virtual reality facemask. BMA: bone marrow aspiration. *reasons for declining were for the first patient a noninterest by this technology and for the second patient a desire to see the gesture and not be distracted by virtual reality.

From December 2019 to December 2020, a total of 105 patients were enrolled (Figure 2). Of these, 19 (18.1%) patients fulfilling the exclusion criteria as well as 17 (16.1%) patients who underwent an iliac crest aspiration were excluded; 1 (1%) patient who removed the mask during procedure was excluded (failure of the procedure), and pain level was not evaluable after procedure. Finally, after age and sex matching, the final cohort included 36 patients, 24 (67%) without VRF (control group) and 12 (33%) wearing a VRF (VRF study group) during BMA.

Patient’s characteristics, BMA indication, and final diagnosis for all patients in the matched cohort are presented in Table 1. Briefly, the median age of patients was 66.7 (IQR: 59.4-76.2) years. More than half of the BMAs were conducted in patients from the internal medicine department (9/36, 25%), from the nephrology department (7/36, 19%), or oncology department (7/36, 19%). Regarding the indication, 15 BMAs (42%) were performed to explore a monoclonal gammopathy, 8 (22%) for an isolated nonregenerative anemia, and 8 (22%) for a bicytopenia or a pancytopenia. Groups did not significantly differ in terms of BMA indication or diagnosis. No complications related to the procedure were recorded.

Among the various relaxing environments, 8 (66%) patients of the VRF group chose the beach, 2 (17%) the forest, and 2 (17%) Zen garden videos. Importantly, the total immersion time recorded in the VRF group was estimated at 15 minutes (IQR 12-15).

Before procedure, anxiety level did not differ between groups, regardless of whoever assessed this parameter (the patient himself or the operator). Thus, in the control group 10/24 (41.7%) patients were considered anxious, and 6/24 (25%) were considered very anxious compared to the 5/12 (41.7%) anxious and 1/12 (8.3%) very anxious patients in the VRF group (*P*=.71). When the patient himself evaluated anxiety level, in the control group 10/24 (45.5%) patients were anxious and 2/24 (9.1%) were very anxious, compared to the 4/12 (33.3%) anxious and 2/12 (16.7%) very anxious patients in VRF group (*P*=.42).

Concerning BMA-associated pain, no difference in NPRS was observed between groups (median NPRS 3.8, IQR 2.0-6.3 vs 3.0, IQR 1.9-3.0; *P*=.09), for the VRF and the control group, respectively).

**Table 1:** Patient’s characteristic, bone marrow aspiration (BMA) indication, and outcomes between wearing a virtual reality mask and not wearing a virtual reality mask during BMA (N=36).

Variable			Matched cohort			*P* value
			Control group (n=24)	VRF^a^ group (n=12)		
**Patient characteristics**						
	Age (years), median (IQR)		66.4 (60.2-75.1)	66.7 (59.1-76.4)	.80	
	**Sex, n (%)**					.99
		Female	11 (46)	6 (50)		
		Male	13 (54)	6 (50)		
**Clinical department, n (%)**						<.001
	Internal medicine		5 (21)	4 (33)		
	Nephrology		5 (21)	2 (17)		
	Oncology		4 (17)	3 (25)		
	Geriatrics		2 (8)	0 (0)		
	Hematology outpatients		1 (4)	0 (0)		
	Surgery units		1 (4)	1 (8)		
	Other departments		6 (25)	2 (17)		
**BMA indication, n (%)**						<.001
	Suspicion monoclonal gammopathy		10 (42)	5 (42)		
	Bicytopenia or pancytopenia		7 (29)	2 (17)		
	Isolated nonregenerative anemia		5 (21)	3 (25)		
	Thrombocytopenia		1 (4)	1 (8)		
	Neutropenia		1 (4)	0 (0)		
	Suspicion metastasic tumors		0 (0)	1 (8)		
**Anxiety level assessed by the operator (%)**						.42
	Nonanxious		8 (33)	6 (50)		
	Anxious		10 (42)	5 (42)		
	Very anxious		6 (25)	1 (8)		
**Anxiety level assessed by the patient (%)**						.71
	Nonanxious		10 (46)	6 (50)		
	Anxious		10 (46)	4 (33)		
	Very anxious		2 (9)	2 (17)		
NPRS^b^ score, median (IQR)			3.0 (1.9-3.0)	3.75 (2.0-6.3)	.09	
**Immersion video, n (%)**						N/A^c^
	Forest		0 (0)	2 (17)		
	Zen garden		0 (0)	2 (17)		
	Beach		0 (0)	8 (66)		
Immersion time, median (IQR)			N/A	15.00 (12.0-15.0)	N/A	

^a^VRF: virtual reality facemask.

^b^NPRS: numerical rating scale score.

^c^N/A: not available.

## Discussion

### Principal Findings

To the best of our knowledge, this study is the first using VRF to try to reduce anxiety and pain during BMA. We did not observe any benefit of the VRF on anxiety levels before or during BMA and pain scores following BMA. We previously showed [4] that a greater level of anxiety before the procedure in patients leads to a greater sensation of pain during BMA as evaluated by the NPRS after the procedure. In this study, the VRF had no significant impact on anxiety or pain. A strength of this prospective study is that preprocedure anxiety levels were not significantly different between the two groups. Therefore, anxiety level did not impact pain assessment as we previously described [4].

The median immersion time with VRF was 15 minutes. Thus, the use of VRF could increase procedure duration, owing to the need to provide explanations to the patient and to the various manipulations for device placement and cleaning. Contrary to our findings, several studies using virtual reality therapy showed positive results in terms of reduction of pain and anxiety during medical procedures [6]. It must be mentioned that results might differ according to patient populations and indications [6].

### Limitations

We acknowledge some limitations. First, the COVID-19 pandemic resulted in a premature arrest of the study, given the risk of contamination between patients using facemask, thus explaining the low number of participants in the VRF group. Therefore, a larger scale and randomized study is needed to confirm our results. Second, sternal BMA is far from being the most common puncture site used worldwide [1]. However, the sternal site is often chosen when BMA is not associated with a bone marrow biopsy. In this study, no complications related to the BMA procedure were recorded [4]. The supine position is far easier for using VRF compared with prone decubitus, which is why our study is focused on sternal BMA. Further studies need to confirm these results for iliac BMA. Third, we acknowledge that anxiety was not assessed with predefined criteria but according to the operator and the patient, as the main objective of this study was the evaluation of VRF on pain. We chose to evaluate anxiety according to our previous paper [4] to allow easier comparison of our results where anxiety was found to be a major predictor of pain during the procedure.

Finally, our cohort included only patients who did not have any BMA previously, which probably explains why patients were usually anxious about this procedure. It would be interesting to conduct a similar study on patients undergoing repeated BMA for chronic malignant hematology disease to see if the technique proves more helpful in this setting.

### Conclusion

This study did not detect any benefit associated with the use of an immersive virtual reality to reduce pain and anxiety associated with sternal BMA in addition to local anesthesia.

## Abbreviations

BMA: bone marrow aspiration
NPRS: numerical rating scale score
VRF: virtual reality facemask
